# Cancer-derived mutation in the OGA stalk domain promotes cell malignancy through dysregulating PDLIM7 and p53

**DOI:** 10.21203/rs.3.rs-2709128/v1

**Published:** 2023-03-20

**Authors:** Chia-Wei Hu, Ao Wang, Dacheng Fan, Matthew Worth, Zhengwei Chen, Junfeng Huang, Jinshan Xie, John Macdonald, Lingjun Li, Jiaoyang Jiang

**Affiliations:** UW-Madison; University of Wisconsin-Madison; University of Wisconsin-Madison; University of Wisconsin-Madison; University of Wisconsin–Madison; University of Wisconsin-Madison; University of Wisconsin-Madison; University of Wisconsin-Madison; University of Wisconsin; Pharmaceutical Sciences Division, School of Pharmacy, University of Wisconsin-Madison

## Abstract

O-GlcNAcase (OGA) is the sole enzyme that hydrolyzes O-GlcNAcylation from thousands of proteins and is dysregulated in many diseases including cancer. However, the substrate recognition and pathogenic mechanisms of OGA remain largely unknown. Here we report the first discovery of a cancer-derived point mutation on the OGA’s non-catalytic stalk domain that aberrantly regulated a small set of OGA-protein interactions and O-GlcNAc hydrolysis in critical cellular processes. We uncovered a novel cancer-promoting mechanism in which the OGA mutant preferentially hydrolyzed the O-GlcNAcylation from modified PDLIM7 and promoted cell malignancy by down-regulating p53 tumor suppressor in different types of cells through transcription inhibition and MDM2-mediated ubiquitination. Our study revealed the OGA deglycosylated PDLIM7 as a novel regulator of p53-MDM2 pathway, offered the first set of direct evidence on OGA substrate recognition beyond its catalytic site, and illuminated new directions to interrogate OGA’s precise role without perturbing global O-GlcNAc homeostasis for biomedical applications.

O-linked β-N-acetylglucosamine (O-GlcNAcylation) is a reversible post-translational modification (PTM) that has been detected on thousands of nucleocytoplasmic proteins (**Extended Data** Fig. 1a)^1,2^. This unique glycosylation dynamically regulates protein activity, stability, localization, ligand binding, PTM crosstalk, among others, playing pivotal roles in modulating protein functions in response to cellular stress and environmental cues^3–5^. Intriguingly, only a single pair of human enzymes governs the dynamic O-GlcNAcylation: O-GlcNAc transferase (OGT) adds GlcNAc moiety to protein Ser/Thr residues, while O-GlcNAcase (OGA) removes the sugar (**Extended Data** Fig. 1a)^6–10^. Both OGT and OGA play critical roles in regulating numerous biological processes and their aberrant functions have been detected in highly deleterious diseases including neurodegeneration^5,11^, metabolic disorders^12,13^, and various types of cancer^5,14,15^. While current research has mainly focused on OGT, the roles and mechanisms of OGA in diseases, especially in cancer, remain challenging to decipher. For instance, different transposon-based mouse studies supported OGA as a cancer-driving gene (according to the Candidate Cancer Gene Database)^16^. However, blocking OGA’s enzymatic activity using catalytic-site inhibitors, an approach that increases the global O-GlcNAcylation, has shown both positive and negative effects on cancer progression^17–24^. These inconsistent findings suggest that OGA orchestrates complex networks in cells, potentially through protein regions beyond its catalytic site. Understanding the functions of OGA’s non-catalytic domains is a prominent step to dissect OGA’s perplexing roles in disease and may facilitate therapeutic interventions.

Human OGA is a multidomain enzyme comprised of an N-terminal catalytic domain with *N*-acetyl glucosaminidase activity, a central stalk domain, and a C-terminal pseudo histone acetyltransferase (pHAT) domain (Fig. 1a)^25^. An interesting study reported that the pHAT domain of OGA regulated protein acetylation and OGT activity^26^. However, the molecular mechanisms underlying the regulatory roles of OGA’s non-catalytic regions remain largely unexplored. While the full-length OGA structure is not available, we and other groups independently reported the truncated human OGA structures using slightly different constructs (e.g., OGA_cryst_) comprising the catalytic domain and most of the stalk domain (Fig. 1a)^27–29^. These structures revealed that human OGA is an unusual dimer with the stalk domain of one monomer covering the catalytic domain of the sister, forming a cleft that can potentially accommodate peptide/protein substrates (Fig. 1a). We further determined a series of OGA_cryst_ structures in complex with distinct O-GlcNAcylated peptides and revealed specific interactions between the peptide substrates and the OGA stalk domain surface residues^30^. These crystal structures shed the first light on OGA’s stalk domain in accommodating diverse peptide substrates. Interestingly, there is a substantial variation between the sequences of human and bacterial OGA stalk domains, implying that the non-catalytic stalk domain has potentially evolved into a unique scaffold to facilitate human OGA interacting with protein partners in the more sophisticated mammalian system. Of note, protein-protein interaction (PPI) surfaces are often mutated in diseases to rewire the functional protein networks^31,32^. Intriguingly, around 43% of the non-silent mutation of human OGA occurred within the stalk domain (according to the Catalogue of Somatic Mutation in Cancer (COSMIC) database)^33^, which is considerably higher than the 31% occurrence in the even longer catalytic domain. This hints at a significant role of OGA’s stalk domain in cancer. However, until now, the molecular and cellular impacts of these OGA stalk domain mutations remain unknown. Understanding how the non-catalytic stalk domain influences the functional association of OGA with other cellular proteins is expected to offer important insights into OGA’s malfunctions in cancer, and the unique substrate recognition mode(s) of OGA that deglycosylates thousands of O-GlcNAcylated proteins without a conserved sequence motif.

PDLIM7 (Enigma) is an O-GlcNAcylated protein belonging to the PDZ and LIM domain (PDLIM) protein family^2,34^. It has been reported to interact with partners of distinct functions, such as receptor^35^, enzyme^36^, transcription factor^37^, tight junction protein^38^, and cytoskeleton^34^, indicating the involvement of PDLIM7 in diverse cellular processes. Interestingly, recent evidence showed that PDLIM7 participates in ubiquitin-proteasome system by cooperating with E3 ubiquitin ligases including CBL-C^39^, PDLIM2^40^ and oncogenic MDM2^41,42^. PDLIM7 was found to bind and stabilize MDM2, thereby promoting the ubiquitination and degradation of its major substrate p53^41^. As one of the most important transcription factors and tumor suppressors, p53 plays multifunctional roles in critical cellular processes in response to stress, and its interaction networks are frequently rewired to fuel tumor cells^43–46^. While around half of all cancers retain wild-type (WT) p53 (ref. 47), their activities are often attenuated by MDM2-induced p53 protein degradation^47–49^. p53-MDM2 axis is an important molecular switch to suppress or promote cancer and is dynamically regulated by different types of PTMs^48,49^. A better understanding of the mechanisms of PTM-regulated p53 network will be critical for therapeutic development. PDLIM7 has been reported as a potential prognostic biomarker in specific carcinomas^50,51^. However, the roles of PTMs on PDLIM7 and their interplay with p53-MDM2 axis in cancer remain largely unknown.

Here, we report that a carcer-derived point mutation on the OGA stalk domain dysregulated a small set of O-GlcNAcylated proteins and PPIs including a p53 subnetwork. Remarkably, we found that OGA mutation on the stalk domain aberrantly and specifically down-regulated the O-GlcNAcylation of PDLIM7 (deglycosylated PDLIM7), which inhibited p53 transcription and promoted p53 degradation via enhanced MDM2-p53 interactions in cells. Moreover, mutation of the OGA stalk domain or the deglycosylated PDLIM7 mutant potentiated cell malignancy. Our studies revealed, for the first time, that OGA’s non-catalytic stalk domain directly regulates specific OGA-protein interactions and O-GlcNAc hydrolysis in a novel cancer promoting mechanism. Our discovery of OGA’s unique role in modulating p53-MDM2 axis through deglycosylation of PDLIM7 not only advances our understanding of the PTM-regulated p53 networks and the protein recognition beyond OGA’s catalytic site, but also opens new avenues to precisely interrogate OGA functions for combating cancer and other diseases.

## Results

### OGA stalk domain mutations promoted malignant cell growth

**Figure 1:**
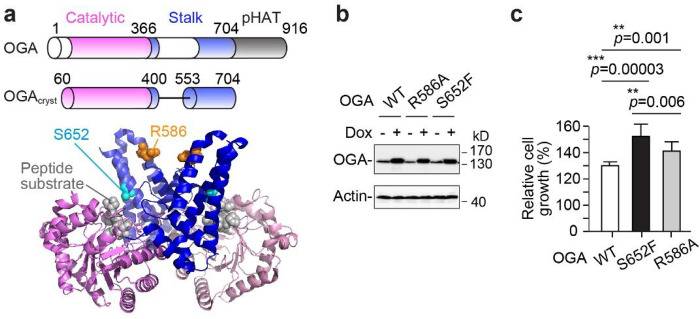
Point mutations on the OGA’s stalk domain promoted aberrant cell growth. **a**, Top: Schematic of OGA and truncated OGA_cryst_ domain structures. The catalytic and stalk domains are shown in pink and blue, respectively. Disordered regions are shown in white. pHAT, pseudo histone acetyltransferase domain. Bottom: The crystal structure of dimeric OGA_cryst_-D175N in complex with an O-GlcNAcylated peptide substrate (PDB: 5VVU)^30^. The coloring is the same as in the schematic (top). **b**, Western blot showing the doxycycline (dox) induced expression of WT OGA and stalk domain mutants in T-REx-293 cells (24 h dox treatment). **c**, The relative cell growth of T-REx-293 cells expressing WT OGA or stalk domain mutant (50 h dox treatment) detected by MTT assay; n = 9. Data are presented as mean ± s.d. **, *p* < 0.01; ***, *p* < 0.001. P-values were determined by one-tailed student’s unpaired t-test.

Hundreds of missense mutations of OGA have been reported in cancer genomic databases (e.g., COSMIC^33^ and cBioPortal for Cancer Genomics^52^). However, little is known about the dysregulation of cancer-derived OGA mutations in cells. As an entry point to address this important question, we investigated the cancer mutations on the OGA stalk domain, a region potentially involved in OGA binding to diverse proteins. We considered the following criteria when selecting OGA variants: 1) classified as a pathogenic mutation (FATHMM score^53^ ≥ 0.7), 2) detected in different types of cancer, 3) most likely retain the structural integrity of OGA protein, and 4) predicted to be the protein-interacting (PPI interface) residue^54^. Accordingly, the S652 residue of OGA (with S652F mutation found in both colon and uterine carcinoma, and a similar mutation S652Y found in liver cancer)^33,52^, located on the stalk domain front surface facing the catalytic pocket of sister OGA monomer, is of particular interest (Fig. 1a). Another predicted protein-interacting residue R586 on the back surface of OGA stalk domain was mutated to Ala (R586A) for comparison (Fig. 1a). To evaluate the functional impacts of OGA mutations in a homogenous cellular background, we exploited the Flp-FRT technique to inducibly express a single copy of Flag-tagged WT OGA or each mutant at the same genomic locus in human embryonic kidney 293 cells (T-REx-293) (ref. 55). This cell system allows rigorous control of desired OGA protein expression at a comparable level for quantitative analysis (Fig. 1b). Meanwhile, we kept the endogenous OGA in these cells to resemble the heterozygous expression commonly observed during cancer development^56^. Notably, we found that both S652F and R586A cells showed significantly increased cell growth compared to WT OGA cells (Fig. 1c **and Extended Data** Fig. 1b). More interestingly, both mutant cells demonstrated substantially higher anchorage-independent growth, a hallmark of cell malignant transformation (**Extended Data** Fig. 1c,d). We further detected the global O-GlcNAcylation of WT OGA and mutant cells using western blot. Compared to WT, we observed differential changes of O-GlcNAcylation on certain proteins in each S652F and R586A cells (**Extended Data** Fig. 1e). To evaluate if the O-GlcNAc changes were induced by the altered intrinsic activity of these OGA mutants, we measured the catalytic efficiency (*k*_cat_/*K*_m_) of each recombinantly purified OGA protein. Using an established fluorogenic assay with 4MU-GlcNAc (primarily binds to the OGA active site) as the substrate^57^, the S652F mutant demonstrated 24% reduced OGA catalytic efficiency, while the catalytic properties of R586A mutant remained largely unperturbed (**Extended Data** Fig. 1f). The western blot and kinetic data indicate that these OGA stalk domain mutants differentially regulate O-GlcNAc hydrolysis of certain proteins in cells, potentially through mechanisms beyond simply modulating the enzymatic activity of OGA. Collectively, these interesting results suggest that a single mutation on the stalk domain altered the substrate specificity of OGA towards a subset of proteins in cells and promoted malignant cell growth.

### Quantitative Proteomic Analyses Discovered A Subset Of Cellular Proteins Dysregulated By OGA Stalk Domain Mutants

To elucidate the molecular mechanisms underlying OGA mutant-induced malignant cell growth, we systematically analyzed the O-GlcNAc profile (O-GlcNAcome), proteome, and interactome of each OGA stalk domain mutant (R586A and S652F), compared to WT OGA, using quantitative LC-MS/MS analysis (**Extended Data** Fig. 2a). To detect the changes of O-GlcNAcylation in cells, we applied metabolic labeling approach with *N*-azidoacetylgalactosamine (GalNAz) that enables the enrichment and detection of O-GlcNAcylated proteins/peptides via click chemistry conjugation with a biotin probe^58^. Consistent with previous reports, cells fed with GalNAz demonstrated higher detection efficiency and minimal non-enzymatic labeling (e.g., S-GlcNAc on Cys residues) compared to other similar methods as evaluated by click chemistry and in-gel fluorescence (**Extended Data** Fig. 2b)^59^. Moreover, the unique, high-intensity diagnostic peaks derived from the biotin-conjugated oxonium ion during MS analysis enabled highly efficient and reliable O-GlcNAc detection through HCD-triggered EThcD fragmentation (**Extended Data** Fig. 2c)^60^. In parallel, the peptide samples without O-GlcNAc enrichment were used for the whole proteome analysis to evaluate protein expression changes. To efficiently capture the OGA protein complexes, especially the interactions related to O-GlcNAcylated substrates, we inducibly expressed “substratetrapping” versions of OGAs with D175N mutation (i.e., Flag-OGA-D175N-HA, Flag-OGA-D175N/S652F-HA, and Flag-OGA-D175N/R586A-HA) that impairs the sugar hydrolysis activity of OGA without compromising its O-GlcNAc binding for interactome study^61,62^. Considering that OGA level varies dramatically in different types of tissues and cells according to the Human Protein Atlas database^63^, we expressed each OGA variant at low and high levels to discover a broad range of OGA binding proteins in cells (**Extended Data** Fig. 2d). The OGA complexes affinity-purified via N-terminal Flag-tag or C-terminal HA-tag were combined to improve the detection coverage of OGA PPIs.

**Figure 2:**
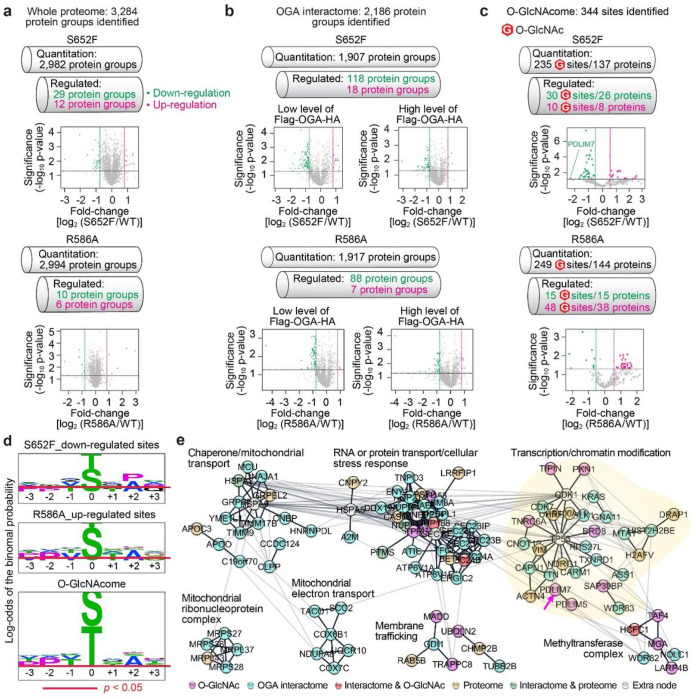
Quantitative proteomic analyses of OGA stalk domain mutants revealed protein network perturbations. **a-c**, Volcano plot and quantitation of the whole proteome (**a**), OGA interactome (**b**), and O-GlcNAcome (**c**). **d**, The semi-consensus sequences of OGA-S652F down-regulated, OGA-R586A up-regulated, and total identified O-GlcNAc sites from O-GlcNAcome analyses. Residues depicted with the residue height over the statistical significance threshold (*p* < 0.05) were considered position-specifically enriched. **e**, The major protein association networks and their representative functions dysregulated by OGA-S652F. Proteins identified from different proteomes were shown as indicated nodes. The light-yellow background highlights the subnetwork associated with p53. PDLIM7 is highlighted by a pink arrow.

We identified a total of 3,284 protein groups from the whole proteome profiling (Fig. 2a). Only dozens of proteins showed significant changes in mutant cells, supporting that the stalk domain mutations did not induce dramatic alteration in protein expression profiles (Fig. 2a **and Supplementary Tables 1,2**. Please see Methods for data and statistical analyses). We found that OGT protein level had no significant change in OGA mutant cells (**Supplementary Table 2**). In the OGA interactome analysis, S652F cells dysregulated 136 OGA PPIs from a total of 2,186 identified proteins, and only 95 aberrant OGA PPIs were detected in R586A cells (Fig. 2b **and Supplementary Tables 3,4**). Interestingly, the stalk domain mutations tend to cause loss rather than gain of OGA-protein interactions (Fig. 2b), which is in good agreement with previous reports on disease mutations that often rewire PPIs by disrupting protein associations^31^. The O-GlcNAc detection in OGA expressing cells was considerably more challenging due to the substantially reduced overall O-GlcNAc intensity (**Extended Data** Fig. 1e). However, we were able to successfully identify 344 unambiguous O-GlcNAc sites (Fig. 2c **and Supplementary Table** 5). The O-GlcNAc stoichiometry was also calculated if the protein was detected in both O-GlcNAcome and proteome. As a result, we identified a list of dysregulated O-GlcNAc sites (proteins): 40 (34) and 63 (53) in S652F and R586A cells, respectively (Fig. 2c **and Supplementary Table 6**). Surprisingly, over 70% of S652F dysregulated O-GlcNAc sites showed decreased O-GlcNAcylation, whereas R586A dysregulated sites showed mostly increased O-GlcNAc modification (Fig. 2c). Further sequence analysis of these two main groups of O-GlcNAcylated peptides (S652F down-regulated and R586A up-regulated O-GlcNAc sites) illustrated mutant-specific divergence of the residues flanking the O-GlcNAc sites (Fig. 2d). Intriguingly, S652F mutant showed a unique enrichment of Pro residue at the + 2 position, whereas the flanking sequences from R586A displayed a similar pattern as the one generated from the entire O-GlcNAcome identified from the same experiments.

Of note, our proteomic analyses showed limited overlaps between S652F and R586A in terms of their dysregulated OGA PPIs and O-GlcNAc sites (**Extended Data** Fig. 3a). To identify the cellular processes that are differentially modulated by these OGA stalk domain mutants, we established the protein networks of OGA S652F or R586A dysregulated proteins (Fig. 2e **and Extended Data** Fig. 3b,c). Both stalk domain mutations affected cellular functions related to malignant transformation, such as transcription^64^. However, the dysregulated proteins by S652F are involved in more diverse biological processes compared to R586A (Fig. 2e **and Extended Data** Fig. 3b). Several S652F dysregulated processes, such as stress response^65^, mitochondria-related functions^66^, and chromatin modification including p53-associated proteins^67^, are essential for regulating cancer cell survival. Taken together, these proteomic studies provided compelling evidence supporting that a single mutation on the OGA’s non-catalytic stalk domain can induce distinct, mutant-specific alterations in OGA-protein interactions and substrate deglycosylation without perturbing the global proteome or O-GlcNAc profile. The bioinformatic analyses further unraveled unique mechanism(s) underlying cancer mutation S652F in cell malignancy.

### Cancer-derived OGA Mutation S652f Aberrantly Deglycosylated PDLIM7, Leading To Significantly Reduced P53 In Cells

**Figure 3:**
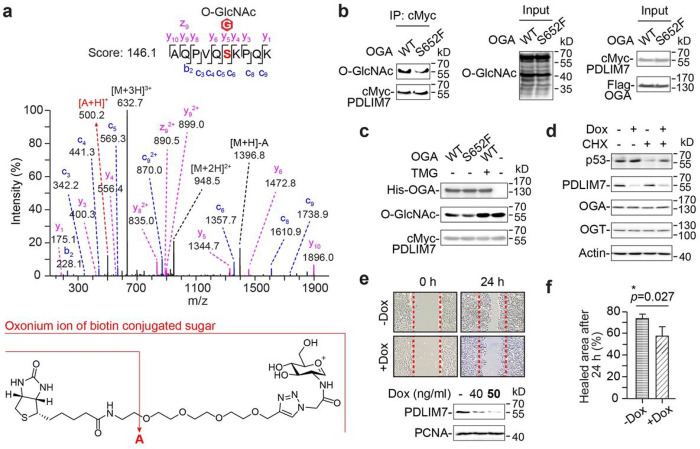
OGA stalk domain mutant S652F dysregulated the O-GlcNAcylation of PDLIM7, a regulator of p53 and cell malignancy. **a**, Top, the MS/MS spectrum of PDLIM7 peptide containing the O-GlcNAcylated S89 residue. Bottom, chemical structure of the hypothetical fragmentation of biotin-conjugated oxonium ion. **b**, Western blot analysis of the O-GlcNAcylation of the immunoprecipitated (IP) cMyc-PDLIM7 from T-REx-293 cells that transiently expressed PDLIM7 for 24 h followed by 27 h induction of Flag-OGA (WT/S652F). O-GlcNAcylation was detected using CTD110.6 antibody. **c**, Western blot analysis of the recombinantly purified cMyc-PDLIM7 and His-OGA from *in vitro* OGA deglycosylation reactions. TMG, thiamet-G. **d**, Western blot analysis of the stability of endogenous p53 in T-REx-293 cells with 24 h of doxycycline (dox)-induced PDLIM7 knockdown. Cycloheximide (CHX) was added to the cells 6 h prior to cell harvesting. **e**, Top, the wound healing of H460 cells with dox-induced PDLIM7 knockdown. Bottom, western blot analysis of the PDLIM7 knockdown in H460 cells (nuclear extracts) treated with dox for 48 h at indicated concentrations. The dox concentration used for the wound healing assay was bolded. **f**, Relative quantitation of the wound healing efficiency from **e** (n = 3). Data are presented as mean ± s.d. *, *p* < 0.05. P-values were determined by one-tailed student’s unpaired t-test.

To further elucidate the molecular mechanisms of cancer-derived OGA mutant S652F in promoting malignancy, we sought to identify the key player from its rewired protein network. As mentioned above, S652F uniquely favored the deglycosylation of O-GlcNAc sites with Pro at + 2 position in cells (Fig. 2d). Among the top dysregulated protein candidates, PDLIM7 possesses such sequence pattern and has been linked to a major S652F rewired network associated with p53 (Fig. 2e). As a critical stress sensor and cell guardian, p53’s expression or function often gets significantly attenuated to fuel cancer progression^43–49^. The protein level of PDLIM7 was reported to affect the abundance of cellular p53 (ref. 41). However, the effects of O-GlcNAc status of PDLIM7 on p53 have not been explored. In this study, we found that OGA S652F significantly down-regulated the O-GlcNAcylation of PDLIM7 at S89 residue (Fig. 2c,3a **and Supplementary Table 6**). The aberrantly decreased O-GlcNAc level on PDLIM7 was validated by immunoprecipitation and western blot using T-REx-293 WT OGA or S652F cells transiently expressing cMyc-tagged PDLIM7 (Fig. 3b). Furthermore, our *in vitro* deglycosylation assay using recombinantly purified WT OGA or S652F protein with O-GlcNAcylated PDLIM7 (gPDLIM7) as the substrate produced consistent results (Fig. 3c). Since the intrinsic activity of S652F protein is lower than WT OGA (**Extended Data** Fig. 1f), the unusually elevated “activity” of S652F toward gPDLIM7 may be attributed to other factors, such as OGA stalk domain associated PPIs. In addition, the dysregulated O-GlcNAc site on PDLIM7 (S89) is remarkably conserved across mammals (**Extended Data** Fig. 4a), implying its important role in regulating PDLIM7 functions in more sophisticated systems.

**Figure 4:**
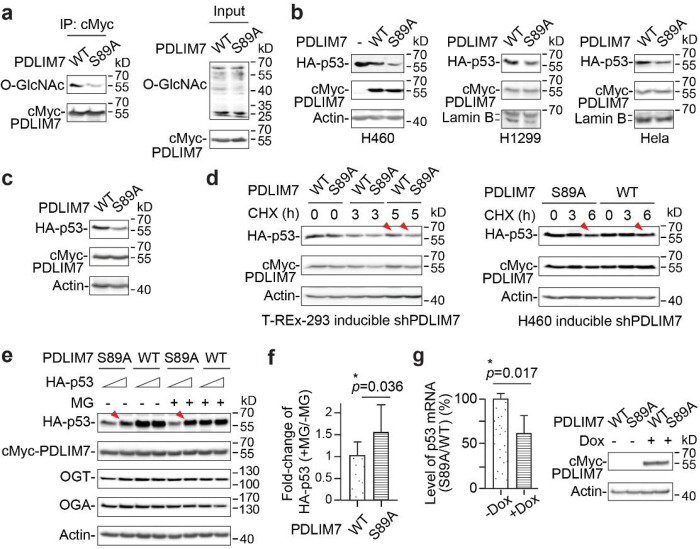
O-GlcNAcylation of PDLIM7 regulated the cellular abundance of p53 at both protein and transcription levels. **a**, Western blot analysis of the O-GlcNAcylation of immunoprecipitated (IP) cMyc-PDLIM7 (WT/S89A) from HEK293 cells after 48 h of expression. O-GlcNAcylation was detected using CTD110.6 antibody. **b**, Western blot analysis of HA-p53 in different cancer cells. For H1299 and Hela cells, p53 was only detected in the nuclear extracts due to the low expression. Cells were transiently co-expressed with cMyc-PDLIM7 (WT/S89A) and HA-p53 for 48 h. **c**, Western blot analysis of HA-p53 in H1299 cells with stable expression of cMyc-PDLIM7 (WT/S89A) and doxycycline (dox)-induced knockdown of endogenous PDLIM7. HA-p53 was expressed similarly as in **b**. Cells were treated with dox 24 h prior to cell harvesting. **d**, Western blot analysis of p53 stability in T-REx-293 (left) and H460 (right) cells with dox-induced knockdown of endogenous PDLIM7. Cells were transiently co-expressed with cMyc-PDLIM7 (WT/S89A) and HA-p53 for 48 h. Dox was added to the cells 24 h post-coexpression. Cycloheximide (CHX) was added at the indicated time points prior to cell harvesting. Red arrows highlight the p53 bands with significant change between WT PDLIM7 and mutant cells. **e**, Western blot analysis of p53 stability in MG132 (MG)-treated H1299 cells with dox-induced cMyc-PDLIM7 (WT/S89A) expression and endogenous PDLIM7 knockdown. HA-p53 expression and dox treatment were performed similarly as in **d**. Red arrows highlight the p53 bands from PDLIM7 S89A cells with or without MG treatment. **f**, The relative protein level of HA-p53 (+MG/−MG) from **e** (n = 4). **g**, Left, real-time qPCR detected the relative level of p53 transcripts from T-REx-293 cells with dox-induced cMyc-PDLIM7 (WT/S89A) expression and endogenous PDLIM7 knockdown. Data were quantified from four independent analyses. Right, western blot analysis of cMyc-PDLIM7 from the same cell culture used for real-time qPCR (left). Cells were treated with dox for 48 h. All quantified data are presented as mean ± s.d. *, *p* < 0.05. P-values were determined by one-tailed student’s paired t-test.

To evaluate the potential impact of PDLIM7 on cellular p53, we monitored the stability of p53 protein in T-REx-293 cells with inducible PDLIM7 knockdown in the presence or absence of the translation inhibitor cycloheximide. We found that PDLIM7 knockdown via its 3’-UTR stabilized the endogenous p53 without affecting the OGT or OGA protein expression (Fig. 3d). To assess whether PDLIM7 could affect cell malignancy, we inducibly knocked down the endogenous PDLIM7 in aggressive lung cancer H460 cells. Indeed, reduced PDLIM7 protein impeded the malignant wound healing of cancer cells (Fig. 3e,f). To further evaluate if the O-GlcNAc status of PDLIM7 was essential for modulating p53 protein level, we generated S89A mutant of cMyc-tagged PDLIM7. Transient expression of this S89A mutant in HEK293 cells showed significantly decreased O-GlcNAcylation compared to WT PDLIM7, supporting that S89 is the major O-GlcNAc site on PDLIM7 (Fig. 4a). We next evaluated the p53 protein levels in cervical cancer Hela cells and two different types of lung cancer cells (H460 and H1299), with transient co-expression of cMyc-tagged PDLIM7 (WT/S89A mutant) and HA-tagged p53. Remarkably, our western blot results showed that the p53 protein level was significantly reduced in all three types of cancer cells expressing deglycosylated S89A mutant compared to the WT PDLIM7 (Fig. 4b). We also obtained similar results in the H1299 cells stably expressing cMyc-tagged PDLIM7 S89A with inducible knockdown of endogenous PDLIM7 via its 3’-UTR (Fig. 4c **and Extended Data** Fig. 4b). All of these results consistently support a previously undiscovered, critical role of PDLIM7 O-GlcNAcylation in regulating p53 abundance in different types of cancer cells.

Since PDLIM7 has been linked to proteasome system^39–42^, we next determined whether the O-GlcNAc level of PDLIM7 affected the cellular stability of p53. T-REx-293 and H460 cells with inducible endogenous PDLIM7 knockdown were co-expressed with cMyc-tagged PDLIM7 (WT/S89A mutant) and HA-tagged p53 followed by the treatment of cycloheximide. In both cell lines, we detected decreased p53 stability in S89A compared to WT PDLIM7 cells (Fig. 4d). Further supporting this, in both H1299 and HEK293 cells inducibly or transiently expressing cMyc-tagged WT PDLIM7/S89A mutant, we found that the treatment of proteasome inhibitor MG132 significantly stabilized p53 protein in S89A compared to WT PDLIM7 cells, while the OGT and OGA proteins remained stable (Fig. 4e,f **and Extended Data** Fig. 4c,d). These results consistently support that the O-GlcNAcylation on the S89 residue of PDLIM7 is important for maintaining the cellular p53 in both cancer and non-cancer cells. To clarify whether this effect was resulted from a crosstalk of O-GlcNAcylation with phosphorylation, we examined the global phosphorylation and PDLIM7 phosphopeptides under similar protein expression conditions using phosphoprotein stain and quantitative LC-MS/MS analysis, respectively. We found that the cells expressing WT PDLIM7 or S89A mutant showed negligible difference in terms of their global and PDLIM7 phosphorylation (**Extended Data** Fig. 5). In line with other LC-MS/MS reports in the PhosphositePlus database^68^, we did not detect any phosphorylation on the S89 residue of PDLIM7. These data all support that the p53 cellular level is mainly regulated by the O-GlcNAcylation rather than the phosphorylation of PDLIM7. In addition, we note that the proteasome inhibition did not fully restore the p53 level in cells with S89A mutant expression compared to WT PDLIM7, implying the potential existence of other mechanisms of modulating p53 in an O-GlcNAcylated PDLIM7-dependent manner (Fig. 4e **and Extended Data** Fig. 4c). Interestingly, PDLIM7 is involved in regulating gene expression^37^. To determine if the O-GlcNAc status of PDLIM7 affected p53 transcription, we quantitatively measured the mRNA level of p53 in T-REx-293 cells with inducible cMyc-tagged PDLIM7 expression and simultaneous knockdown of endogenous PDLIM7. Strikingly, our real-time qPCR experiments detected significantly lower transcripts of p53 in S89A compared to WT PDLIM7 cells (Fig. 4g), indicating a dual regulatory role of O-GlcNAcylated PDLIM7 in modulating p53 at both protein and transcription levels.

### The O-GlcNAc status of PDLIM7 regulates p53 ubiquitination by modulating the interactions of PDLIM7 with MDM2-p53 complex in cells

**Figure 5:**
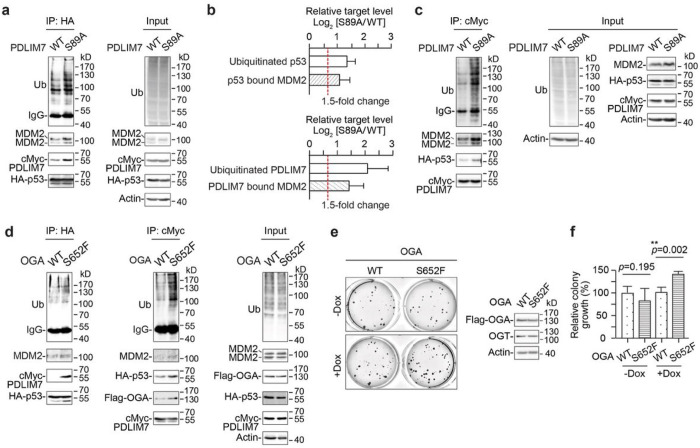
Deglycosylated PDLIM7 (S89A) promoted the ubiquitination of p53 and MDM2-p53 interaction in a similar manner as OGA stalk domain mutant S652F. **a**, Western blot analysis of the ubiquitination (Ub) of the immunoprecipitated (IP) HA-p53 and its complexes with MDM2 and cMyc-PDLIM7 (WT/S89A) from HEK293 cells. Cells were transiently expressed with cMyc-PDLIM7 (WT/S89A), HA-p53, and ubiquitin for 48 h prior to MG132 (MG) treatment. **b**, Top, relative quantitation of p53 ubiquitination and its bound MDM2 in a (n = 3). Bottom, relative quantitation of PDLIM7 ubiquitination and its bound MDM2 in **c** (n = 4). **c**, Western blot analysis of the ubiquitination of the immunoprecipitated cMyc-PDLIM7 (WT/S89A) and its complexes with MDM2 and p53 from T-REx-293 cells with doxycycline (dox)-induced knockdown of endogenous PDLIM7. The co-expression of cMyc-PDLIM7 (WT/S89A), HA-p53 and ubiquitin, as well as the MG treatment, were performed similarly as in **a**. Cells were treated with dox 27 h prior to cell harvesting. **d**, Reciprocal IPs of HA-p53 or cMyc-PDLIM7 complexes from T-REx-293 cells with dox-induced Flag-OGA (WT/S652F) expression and endogenous OGA knockdown. The co-expression of cMyc-PDLIM7 (WT), HA-p53 and ubiquitin, and the treatment of dox and MG were performed similarly as in **c**. **e**, Left, soft agar assay of T-REx-293 cells with dox-induced Flag-OGA (WT/S652F) expression and endogenous OGA knockdown. Right, western blot analysis of the expressions of Flag-OGA and OGT from the same cell culture used in soft agar assay (left). **f**, Relative quantitation of the colony growth from **e**(n = 3). All quantified data are presented as mean ± s.d. **, *p* < 0.01. P-values were determined by one-tailed student’s paired t-test.

PDLIM7 has been reported to interact with E3 ubiquitin ligase MDM2, a well-known ubiquitination and degradation regulator of p53 (ref. 41). We next investigated the effects of PDLIM7 O-GlcNAcylation on p53-MDM2 association and p53 ubiquitination. In HEK293 cells co-expressing cMyc-tagged PDLIM7 (WT/S89A mutant), HA-tagged p53 and ubiquitin, immunoprecipitated p53 showed significantly increased ubiquitination and MDM2 binding, along with enhanced association of p53 with PDLIM7 S89A mutant compared to WT PDLIM7 (Fig. 5a, **top of 5b**). Interestingly, we detected consistent change in lung cancer H1299 cells stably expressing cMyc-tagged PDLIM7 S89A with inducible knockdown of endogenous PDLIM7 (**Extended Data** Fig. 6a), suggesting that deglycosylated PDLIM7 promoted MDM2-induced p53 ubiquitination and degradation. It is worth mentioning that no obvious difference in the O-GlcNAcylation of p53 was detected between H1299 cells with inducible expression of WT PDLIM7 and S89A mutant, despite the generally weak O-GlcNAcylation on p53 (**Extended Data** Fig. 6b). The positive correlation between deglycosylated PDLIM7 (i.e., S89A) and p53 ubiquitination may stem from the enhanced interactions between PDLIM7 S89A and MDM2 compared to the WT PDLIM7, which may also contribute to the elevated ubiquitination of PDLIM7 S89A itself (**bottom of** Fig. 5b, 5c). In all these immunoprecipitation experiments, we noticed a moderate increase of MDM2 level in PDLIM7 S89A expressing cells, indicating that the deglycosylated PDLIM7 might further stabilize MDM2 compared to its O-GlcNAcylated counterpart. However, the change of MDM2 protein level did not achieve statistical significance (**Extended Data** Fig. 6c). To assess if OGA stalk domain modulated p53 ubiquitination by regulating the O-GlcNAcylation of PDLIM7, we performed similar analyses using T-REx-293 cells with inducible knockdown of endogenous OGA via its 3’-UTR and simultaneous expression of WT OGA or S652F mutant. Remarkably, the immunoprecipitated p53 complexes from OGA S652F cells showed consistently increased p53 ubiquitination and p53-MDM2 association compared to WT OGA (**left of** Fig. 5d **and Extended Data** Fig. 6d). OGA S652F cells also showed substantially increased ubiquitination and MDM2 binding of cMyc-tagged PDLIM7 (**right of** Fig. 5d). Furthermore, we detected enhanced interaction between OGA S652F mutant and PDLIM7 in cells (**right of** Fig. 5d), in line with our detection of significantly reduced O-GlcNAcylation on PDLIM7 in OGA S652F compared to WT OGA cells from above mentioned proteomic analysis and *in vitro* experiments (Fig. 2c,3c **and Supplementary Table 6**). Notably, the same OGA S652F cell line also demonstrated abnormally higher malignant anchorage-independent growth than WT OGA cells without perturbing the cellular level of OGT protein (Fig. 5e,f). Taken together, these data strongly support that the cancer-derived OGA mutation S652F aberrantly deglycosylated PDLIM7 and promoted p53 ubiquitination and degradation by stabilizing p53-MDM2 interactions in cells.

**Figure 6:**
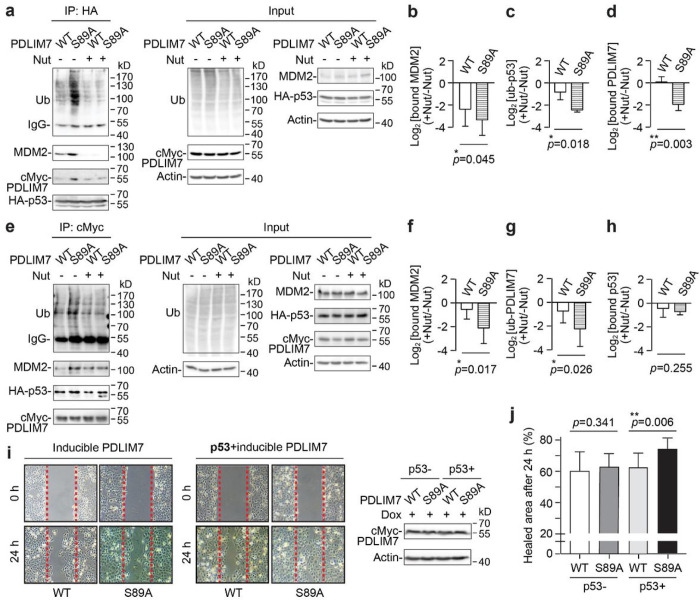
Deglycosylated PDLIM7 (S89A) regulated p53 ubiquitination and cell malignancy in an MDM2-dependent manner. **a**, Western blot analysis of the immunoprecipitated (IP) HA-p53 complex from HEK293 cells treated with Nutlin-3a (Nut). Cells were transiently expressed with cMyc-PDLIM7 (WT/S89A), HA-p53, and ubiquitin (Ub) for 48 h followed by 12 h of Nut and 3 h of MG132 (MG) treatments. **b-d**, Relative quantitation (+Nut/−Nut) of p53 bound MDM2 (**b**), p53 ubiquitination (**c**), and p53 bound PDLIM7 (WT/S89A) (d) from **a**(n = 3). **e**, Western blot analysis of the immunoprecipitated cMyc-PDLIM7 complex from Nut-treated T-REx-293 cells with doxycycline (dox)-induced knockdown of endogenous PDLIM7. The co-expression of PDLIM7 (WT/S89A), p53 and ubiquitin, and the treatment of Nut and MG were performed similarly as in **a**. Dox was added to the cells 24 h post-coexpression. **f-h**, Relative quantitation (+Nut/−Nut) of PDLIM7-bound MDM2 (**f**), PDLIM7 ubiquitination (**g**), and PDLIM7 (WT/S89A) bound p53 (**h**) from **e** (n = 3). **i**, Left, wound healing assay of H1299 cells (+/− HA-p53) with dox-induced cMyc-PDLIM7 (WT/S89A) expression and endogenous PDLIM7 knockdown. Right, western blot analysis of PDLIM7 from the same cell culture used in wound healing assay (left). **j**, Relative quantitation of the wound healing efficiency from **i**; p53- (n = 6); p53+ (n = 8). All quantified data are presented as mean ± s.d. *, *p* < 0.05; **, *p*< 0.01. P-values were determined by one-tailed student’s paired (**b-d, f-h**) or unpaired (j) t-test.

We further investigated the unusual binding of deglycosylated PDLIM7 with MDM2 and tested if the PDLIM7 O-GlcNAcylation-regulated p53 ubiquitination was MDM2 dependent. We employed HEK293 or T-REx-293 cells with inducible knockdown of endogenous PDLIM7 to co-express cMyc-tagged PDLIM7, HA-tagged p53 and ubiquitin as described above. Nutlin-3a, a widely used inhibitor that specifically targets the p53 binding site on MDM2, was applied to block the p53-MDM2 interaction in cells^69^. The ubiquitination of p53, as well as the association of p53, MDM2, and PDLIM7 were analyzed by reciprocal immunoprecipitation and western blot. As expected, Nutlin-3a efficiently blocked the p53-MDM2 interaction in both WT PDLIM7 and S89A mutant cells (Fig. 6a,b). Intriguingly, we found that Nutlin-3a eliminated the augmented ubiquitination of p53 in deglycosylated PDLIM7 S89A mutant compared to WT PDLIM7 cells (Fig. 6a,c). The aberrantly enhanced association of p53 with PDLIM7 S89A was also disrupted by Nutlin-3a (Fig. 6a,d). Surprisingly, Nutlin-3a also significantly reduced the association of MDM2 with PDLIM7 S89A, as well as its ubiquitination, while the impact on WT PDLIM7 was negligible (Fig. 6e–g). Moreover, in the immunoprecipitated PDLIM7 complex, we noted that Nutlin-3a did not fully block the PDLIM7 (WT or S89A mutant) binding with p53, implying the potential existence of MDM2-independent interaction of PDLIM7 and p53 (Fig. 6e,h). Although other types of complexes may co-exist in cells, these findings in general support that the deglycosylated PDLIM7 modulated p53 ubiquitination primarily in an MDM2-dependent way. To further dissect the mechanisms of PDLIM7 deglycosylation in promoting cell malignancy, we exploited the p53-null lung cancer H1299 cells with and without HA-tagged p53 stable expression to inducibly knockdown endogenous PDLIM7 and simultaneously express cMyc-tagged PDLIM7 (WT/S89A mutant). We performed wound healing assay to evaluate their malignant cell migration. Interestingly, significantly faster cell migration of deglycosylated PDLIM7 S89A cells than WT PDLIM7 cells was detected in a p53-dependent manner (Fig. 6i,j **and Extended Data** Fig. 6e), strongly supporting that the deglycosylated PDLIM7 prompted cell malignancy through p53 pathways.

## Discussion

Recent years have seen impressive progress on O-GlcNAc detection with a large repertoire of O-GlcNAcylated proteins identified from diverse stress conditions^2^. However, the molecular mechanisms of how OGA discerns various protein substrates in response to different stimuli remain elusive. Our previous studies on OGA structures in complex with distinct O-GlcNAcylated peptide substrates indicate that the stalk domain of OGA may contribute to its substrate recognition^30^. In this study, we report the first discovery of cancer-derived mutation (S652F) on the OGA stalk domain that potentiates malignant phenotypes and the underlying molecular pathways. Our systematic analyses of OGA interactome and O-GlcNAc profiling revealed that a single mutation on the solvent-exposed surface of stalk domain altered OGA interactions with a subset of substrates and non-substrate proteins involved in regulating cell malignant processes. Surprisingly, the OGA stalk domain S652F mutant with lower intrinsic enzymatic activity more efficiently removed the O-GlcNAcylation of PDLIM7 (and potentially a few other protein substrates) than WT OGA *in vitro* and in cells. This “enhanced” activity toward PDLIM7 seems to be specific for S652F mutant because OGA R586A mutant on the same stalk domain did not display this “enhanced” activity (**Supplementary Table 6**). Our following biochemical and cellular experiments strongly support that the interactions between OGA stalk domain and protein substrates/non-substrates could be a crucial factor in modulating OGA functions and O-GlcNAc dynamics in response to cellular stress and environmental cues.

O-GlcNAc homeostasis is critical for health maintenance and is frequently dysregulated in various types of cancer^14,15^. Tremendous efforts have been attempted to correlate OGA, the sole enzyme that removes O-GlcNAc modifications from numerous proteins, with tumor grades, prognosis/patient survival, and anti-cancer treatment^70^. However, the varied outcomes from different cancer studies, potentially due to the feedback response and undesired side effects of global O-GlcNAc perturbation, made it difficult to define the precise role of OGA in malignant cell progression^17–24^. Our studies on a cancer-derived single mutation (S652F) of OGA provided the first set of direct evidence supporting the non-catalytic stalk domain in regulating OGA-protein interactions and site-specific deglycosylation without perturbing the global proteome or O-GlcNAcome. Our results uncovered a novel mechanism in which the OGA S652F mutation aberrantly removes O-GlcNAcylation from PDLIM7 (deglycosylated PDLIM7), promoting p53 ubiquitination and degradation by enhancing the interactions with MDM2. In addition, deglycosylated PDLIM7 inhibits p53 gene expression. Both mechanisms effectively reduce p53 in different types of cells, explaining at least in part the pathogenic roles of OGA S652F in carcinoma. Our novel findings strongly support that the cancer-derived mutations on the OGA stalk domain (and potentially other non-catalytic regions) can rewire OGA-protein networks and reprogram the associated cellular machineries. A better understanding of protein functional modules dysregulated by OGA mutations may illuminate new directions to dissect and intervene the manifold roles of OGA in biology and disease for more precise control.

P53-MDM2 complex is the central hub of diverse cellular functions, in which the activity of p53 is mainly controlled by MDM2 triggered ubiquitination and proteasomal degradation^44,45, 47–49^. Many cancers possess low or no p53 activity^47^ and may also have MDM2 amplification^49,71^. Hence, stabilizing/re-activating p53 and/or inhibiting MDM2 have been a main focus in cancer therapeutic development^46,72,73^. While exciting progress has been made, many challenges limit their clinical applications^72,73^. One major obstacle is the remarkably sophisticated, highly dynamic, PTM regulated network of p53-MDM2 that integrates various factors to precisely control the cellular functions upon stress or stimuli^44,45,48,49^. Simply targeting p53 or MDM2 itself may break the homeostasis of many other essential systems. Thus, a more complete understanding of their regulatory mechanisms is much needed to improve the therapeutic efficacy and safety of targeting p53-MDM2 axis. As a co-factor of MDM2, PDLIM7 has been proposed as a potential diagnostic biomarker in carcinoma. However, the precise roles of PDLIM7, especially its PTMs, in shaping cell malignancy have not been reported. Our analyses of OGA cancer mutant dysregulation have led to the discovery of aberrant deglycosylation of PDLIM7 which profoundly promoted p53-MDM2 association, p53 ubiquitination, and malignant cell progression. In a further support of this novel mechanism, the augmented p53 ubiquitination induced by deglycosylated PDLIM7 was eliminated in HEK293T cells with inactive p53 (**Extended Data** Fig. 6f), in which the p53 and MDM2 functions are blocked by SV40 large-T antigen^74,75^. Moreover, we found that the inhibitor of MDM2-p53 interaction, Nutlin-3a, also disrupted the aberrant association of MDM2 with deglycosylated PDLIM7 (S89A) but displayed negligible impact on WT PDLIM7-MDM2 interaction (Fig. 6e,f). The differential responses to Nutlin-3a between WT PDLIM7 and S89A mutant cells indicate that PDLIM7 may be able to adopt distinct binding modes/sites on MDM2 or MDM2-p53 complex, depending on the O-GlcNAc status. This interesting observation requires further investigation. In summary, all the evidence mentioned above highlights the O-GlcNAc status of PDLIM7 as a novel regulator of p53-MDM2 functions, uncovering a potential new avenue of targeting OGA-PDLIM7 interaction for therapeutic interventions.

## Methods

### Chemical and Antibodies

GalNAz, Ac_4_GalNAz, Ac_4_GlcNAz, Ac_3_6AzGlcNAc, GlcNAz, 6AzGlcNAc, and thiamet-G were synthesized and characterized to be > 95% pure as previously reported^76–79^. Commonly used chemicals and solvents were purchased from MilliporeSigma or Thermo Fisher. Other chemicals and antibodies are listed in **Supplementary Table 7**.

### Molecular Cloning

To generate pgPDLIM7 for protein purification, OGT and cMyc-tagged PDLIM7 were subcloned into pETDUET (MilliporeSigma) in which PDLIM7 was fused with an N-terminal His_6_-tag. The shRNAs targeting the 3’-UTR of OGA (TRCN0000285410) or PDLIM7 (TRCN0000350814) were selected from the Genetic Perturbation Platform (Broad Institute) and cloned into the EZ-Tet-pLKO-Puro (Addgene #85966) to generate EZ-Tet-pLKO-Puro-shOGA or EZ-Tet-pLKO-Puro-shPDLIM7 as previously described^80^. Human full-length OGA expression plasmid pcDNA5-FRT-TO-Flag-OGA-HA was generated by PCR amplification of OGA using primers encoding the N-terminal Flag_3_ (DYKDHDGDYKDHDIDYKDDDDK)-tag and C-terminal HA (YPYDVPDYA)-tag, and subcloned into the pcDNA5-FRT-TO (Addgene #40998). pcDNA5-FRT-TO-Flag-OGA was generated similarly using primers encoding the Flag_3_-tag. Human full-length PDLIM7 expression plasmid pcDNA3.1-PDLIM7 was generated by PCR amplification of PDLIM7 from PDLIM7_pLX304 (Harvard PlasmID #HsCD00411962) and subcloned into pcDNA3.1 fused with a C-terminal cMyc (EQKLISEEDL)-His_6_-tag (Addgene #64281). pcDNA5-FRT-TO-PDLIM7-cMyc, pLenti-PDLIM7-cMyc, and Tet-O-PDLIM7-cMyc-Hygro were similarly generated from pcDNA5-FRT-TO, pLenti (a kind gift from Dr. Meyer Jackson), and Tet-O-Hygro (Addgene #117271), respectively. Human HA-tagged p53 expression plasmid pcDNA3-p53 was purchased from Addgene (#69003), and subcloned into pLenti to generate pLenti-p53.

All plasmids encoding OGA and PDLIM7 mutants were generated by the QuikChange II XL Site-Directed Mutagenesis Kit (Agilent) according to manufacturer’s instructions, using the wild-type (WT) PDLIM7 or OGA plasmids as the DNA templates. All the constructs generated in this study were verified by DNA sequencing. All the primers and plasmids used in this study are listed in **Supplementary Table 8 and 9,** respectively.

### Cell Lines, Cell Culture, and Doxycycline Induction

The Flp-In T-REx-293 (T-REx-293) cell line was purchased from Invitrogen (#R78007). HEK293, HEK293T, Hela, NCI-H1299, and NCI-H460 cell lines were obtained from American Type Culture Collection (ATCC). The stable cell lines used in this study were generated from T-REx-293, H1299, or H460 cells (**Supplementary Table 10**). All cells were maintained in 5% CO_2_ at 37 °C. H1299 and the derived stable cell lines were cultured in RPMI-1640 media (Corning) supplemented with 10% fetal bovine serum (FBS, MilliporeSigma #F0926) and 0.8% penicillin-streptomycin unless otherwise indicated. Other cell lines were cultured in Dulbecco’s Modified Eagle Medium (DMEM, Corning) supplemented with 10% FBS and 0.8% penicillin-streptomycin. To induce expression or knockdown of protein target in the stable cell lines, cells were treated with doxycycline ranging from 2.5–60 ng/mL for the indicated time.

### Transfection, Lentiviral Particle Production, and Generation of Stable Cell Lines

For transient transfection of HEK293T, HEK293, T-REx-293, and T-REx-293 derived stable cell lines, cells were seeded in 6-well plate 22–24 h prior to transfection (around 70% confluency). The plasmids were delivered into the cells using calcium phosphate as previously reported^81^. Transient transfection of Hela, H460, H1299, and their derived stable cell lines were performed by Trans IT-X2 (Mirus) or Trans IT-2020 (Mirus) following manufacturer’s instructions. Cells were seeded in 6-well plate 20–24 h prior to transfection (around 80–90% confluency). All cells were cultured in antibiotic-free media during the transfection process. In general, 0.5–4 μg DNA of each plasmid was applied for transfection in each well. Doxycycline was added 24 h post-transfection if the induction was needed. Cells were washed by PBS, harvested by scrapping, and stored at −80 °C until use.

For producing lentiviral particles, HEK293T cells were co-transfected with the plasmid encoding target cDNA or shRNA, psPAX2 (Addgene #12260), and pCMV-VSV-G (Addgene #8454) (**Supplementary Table 9**). Lentivirus were harvested at 48 and 72 h post-transfection and stored at −80 °C until use.

To generate stable cell lines with Flp-In T-RΕx inducible expression, T-REx-293 cells were co-transfected with plasmids encoding the target genes and pOG44 (Invitrogen #V600520) at a ratio of around 1:10 using calcium phosphate as mentioned above (**Supplementary Table 9**). Other stable cell lines were generated by lentiviral transduction with 0.08 μg/mL hexadimethrine bromide for 48 h. Cells were then replated in 10-cm plate and screened for 2–3 weeks with antibiotics-containing media.

### Western Blot

To prepare the whole cell lysates, cell pellets were lysed in RIPA buffer (50 mM Tris pH 8.0, 150 mM NaCl, 0.2 mM EDTA, 0.1% SDS, 0.5% sodium deoxycholate, and 1% NP-40). To prepare the nuclear extracts, cell pellets were first lysed in PBS containing 0.1% NP-40 followed by centrifugation at 10,000 g for 30 min at 4 °C. The remaining pellets were then lysed in nuclear extraction buffer (50 mM Tris pH 8.0, 400 mM NaCl, 20% glycerol, 0.5% sodium deoxycholate, 1% NP-40, and 0.5% SDS). All buffers contain protease inhibitor cocktail (MilliporeSigma). For O-GlcNAcylation detection, 10 μM thiamet-G was included in the buffer during cell lysis. Cell debris was removed by centrifugation at 16,000 g at 4 °C for 20 min. Protein concentration was measured by BCA protein assay kit (Pierce). Cell lysates were separated by SDS-PAGE and transferred onto a nitrocellulose membrane using iBlot (Life Technology). The membrane was then blocked with 0.9% bovine serum albumin followed by primary antibody hybridization overnight at 4 °C. Secondary antibody was applied to the blot with 1 h incubation at room temperature (r.t). The blot was developed by ECL substrate (Azure Biosystem or Bio-Rad). Signal was detected on C600 imaging system (Azure Biosystem) using chemiluminescent mode. Relative quantitation of the proteins was conducted by the ImageStudio Lite software (v 5.2, LI-COR). The overall intensity of the detected target was normalized to the corresponding loading control protein (actin for the whole cell lysates, lamin B1 or PCNA for nuclear extracts) in each sample.

### In-Gel Fluorescence

For metabolic labeling, cells were seeded in 6-well plates to reach 70% confluency. After around 20 h of seeding, the growth media was replaced with low-glucose, antibiotic-free DMEM (0.5 g/L glucose, 10% FBS) containing different sugar analogues (200 μM for Ac_4_GalNAz, Ac_4_GlcNAz, and Ac_3_6AzGlcNAc; 2 mM for GalNAz, GlcNAz, and 6AzGlcNAc). After 24 h incubation, cells were washed by PBS, harvested by scrapping, and stored at −80 °C until use. Cell pellets were lysed in NP-40 buffer (1% NP-40, 150 mM NaCl, and 50 mM HEPES pH 7.5). Cell debris was removed by centrifugation at 16,000 g at 4 °C for 20 min. Protein concentration was measured as described above. 35 μg lysates were diluted to 1 μg/μL by 20 mM HEPES pH 7.5. The freshly prepared click chemistry reagent mix (1 mM CuSO_4_, 5 mM Tris(3-hydroxypropyltriazolylmethyl)amine (THPTA), 50 μM uor 488-alkyne (MilliporeSigma), and 7.5 mM sodium ascorbate) was then added to each sample and incubated for 1 h in the dark. Samples were precipitated by MeOH overnight at −80 °C and re-dissolved in 4% SDS. For *in vitro* non-enzymatic labeling, lysates similarly prepared from the HEK293 cells without sugar treatment were incubated with each sugar analogue (200 μM) at 37 °C for 2 h followed by click chemistry and protein precipitation. The re-solubilized proteins were separated by SDS-PAGE and detected by fluorescence scanning. Coomassie blue staining was then applied to obtain the total protein loading. All the imaging was performed on C600 imaging system.

### Co-Immunoprecipitation (co-IP)

For OGA interactome analysis, T-REx-293 cells with inducible OGA-D175N variant were cultured in 15-cm plate at 80% confluency for 17–18 h followed by 8 h of doxycycline induction. Cells were then washed by PBS, harvested by scrapping, snap-frozen in liquid N_2_, and stored at −80 °C until use. The cell pellet was lysed in IP buffer (1% NP-40, 50 mM HEPES pH 7.5, 150 mM NaCl, 0.1% sodium deoxycholate). Protein concentration was determined as mentioned above. Lysates were then diluted to 1 μg/μL by PBS. All buffers contained protease inhibitor cocktail. The affinity purification of Flag-, HA-, or cMyc-tagged protein complexes were performed as previously reported using anti-Flag (M2, MilliporeSigma), anti-HA (Pierce) or anti-cMyc agarose (Pierce), respectively, at 4 °C for 16–19 h with gentle rotation^82^. The resins were gently washed three times with 20 mM Tris-HCl pH 7.5, 150 mM NaCl, and 0.05% NP-40 followed by washing once with 20 mM Tris-HCl pH 7.5 and 150 mM NaCl. The resin bound proteins were eluted by 0.1 M glycine pH 2.6 containing 0.5% NP-40, and immediately neutralized by 1 M NH_4_HCO_3_ followed by MeOH precipitation overnight at −80 °C. Protein pellets were washed with MeOH and solubilized in 8 M urea/0.1 M triethylammonium bicarbonate (TEAB) with rotation and sonication. Protein solution was then subjected to the sample preparation for proteomic analysis (please see “Sample Preparation for Proteomic Analysis” section). Sample for PDLIM7 phosphopeptide analysis was similarly prepared using T-REx-293 cells with inducible knockdown of endogenous PDLIM7 and co-transfection with HA-tagged p53, ubiquitin, and cMyc-tagged WT PDLIM7 or mutant. IP samples for western blot analysis were prepared similarly as above with the following modifications: 10 μM thiamet-G was included in the buffer during cell lysis and affinity purification to retain the protein O-GlcNAcylation if needed; 10 mM iodoacetamide was included in the buffer during cell lysis for ubiquitination detection; 1/20 of the total lysates for affinity purification was used as the input control; three-time wash was applied to affinity purification for O-GlcNAcylation detection; the resin bound proteins were eluted twice by incubating with two-fold SDS-loading buffer at 35 °C for 15 min. For the second elution, resins were boiled for 5 min prior to centrifugation. All protein samples were subjected to SDS-PAGE for western blot analysis. Relative quantitation of the protein was performed using ImageStudio Lite software. The intensity of detected target was normalized to the intensity of corresponding actin loading control or the total affinity purified tagged proteins.

### Biotin Conjugation of O-GlcNAcylated Proteins for O-GlcNAcome Analysis

T-REx-293 cells with inducible Flag-tagged WT OGA or mutants were seeded at 6×10^6^ cells/plate in 15-cm dish for 27–28 h. The growth medium was then changed to low-glucose (0.5 g/L), antibiotic-free DMEM supplemented with 10% FBS and 2.5 mM GalNAz. Doxycycline was also added to the cells for Flag-tagged OGA induction. After 24 h incubation, cells were washed by PBS, harvested by scrapping, snap-frozen in liquid N_2_, and stored at −80 °C until use. Cell pellets were first lysed in NP-40 buffer for 1 h followed by centrifugation (16,000 g, 20 min, 4 °C). The remaining pellets were further lysed in SDC buffer (0.5% sodium deoxycholate, 400 mM NaCl, 20% glycerol, and 50 mM HEPES pH 7.5) followed by centrifugation to remove cell debris. All buffers contained protease inhibitor cocktail and 10 μM thiamet-G. Protein concentration was determined as mentioned above. 10 and 1 mg cell lysates from the first and the second lysis step were diluted to 2.5 and 1 μg/μL, respectively, by 50 mM HEPES pH 7.5. Freshly prepared click chemistry reagent mix (5 mM THPTA, 1 mM CuSO_4_, 100 uM Biotin-PEG_4_-alkyne, and 7.5 mM ascorbate) was then added to each lysate sample followed by 2 h incubation at r.t with gentle rotation. Lysates from the same cell sample were then combined for MeOH precipitation and re-solubilization as mentioned above. The re-solubilized proteins were subjected to O-GlcNAcome and whole proteome analyses as described in “Sample Preparation for Proteomic Analysis”.

### Sample Preparation for Proteomic Analysis

#### Protein Digestion

Proteins were first reduced by 10 mM dithiothreitol for 30 min at r.t. followed by alkylation with 55 mM iodoacetamide in the dark at r.t. for 30 min. The protein solution was then seven-fold diluted using 25 mM TEAB and digested by trypsin/Lys-C mix (100:1, protein/protease, w/w, Promega) at r.t. for 20 h. Protease activity was quenched by 0.5% formic acid (FA) followed by SDB-XC StageTip desalting as previously reported^83^. The final peptide samples were dried by vacuum centrifugation and stored at −80 °C until use.

#### O-GlcNAcylated Peptide Enrichment

The dried peptides were resuspended in 0.1 M TEAB with incubation and sonication. The peptide concentration was estimated by BCA assay. For whole proteome analysis, 10 μg peptides from each condition were directly subjected to dimethyl labeling (please see “Dimethyl Labeling” section). For O-GlcNAcome analysis, the same amount of peptides from each condition was diluted to approximately 2 mg/mL with PBS. The enrichment of biotin-conjugated peptides was performed using the reported DiDBiT method with slight modifications^84^. Brie y, peptide solution was mixed with NeutrAvidin agarose resin (Life Technology) for 3 h at r.t. with rotation. The resins were pelleted by centrifugation (1,000 g, 5 min) to remove unbound peptides and washed once with 1 mL PBS, four times with 5% ACN/PBS, and once with 1 mL HPLC water. The biotinylated peptides were then eluted three times by 80% ACN/0.3% FA. For the last elution, resins were heated at 80 °C for 5 min prior to centrifugation. The combined eluents were dried by vacuum centrifugation and stored at −80 °C until dimethyl labeling.

#### Dimethyl Labeling

Tryptic peptide samples were redissolved in 100 μL of 0.1 M TEAB solution. The labeling was conducted as previously reported^85^. Briefly, every 100 μL peptide sample from WT OGA, R586A, and S652F mutants were added with 6 μL of 4% formaldehyde-H_2_ (L), 4% formaldehyde-D_2_ (M), and 4% formaldehyde-^13^CD_2_O (H), respectively. Samples were then mixed with 6 μL of freshly prepared sodium cyanoborohydride (for “L” and “M” labeling) or sodium cyanoborodeuteride (for “H” labeling). Each sample was vigorously mixed and incubated at r.t. for 60 min with rotation. The reaction was quenched by adding 1% ammonium hydroxide and 10% FA. The L-, M- and H-labeled samples were then combined and subjected to SDB-XC StageTip desalting. The final peptide samples were dried by vacuum centrifugation and stored at −80 °C until peptide fractionation.

#### Peptide Fractionation

Peptide fractionation was conducted by SDB-RPS StageTip as previously reported^86,87^. Peptides were eluted with the following buffers: 60 mM ammonium acetate/20% ACN/0.5% FA, 100 mM ammonium acetate/30% ACN/0.5% FA, 150 mM ammonium acetate/80% ACN/0.5% FA, and 5% ammonium hydroxide/80% ACN. For O-GlcNAcome analysis, two additional fractions were collected using 30 mM ammonium acetate/10% ACN/0.5% FA and 80 mM ammonium acetate/60% ACN/0.5% FA. For whole proteome analysis, an additional fraction was collected using 80 mM ammonium acetate/60% ACN/0.5% FA.

### NanoLC-MS/MS Analysis

Peptides were resuspended in 0.1% FA and analyzed on either a Thermo Scientific Orbitrap Fusion Lumos Tribrid mass spectrometer (for O-GlcNAcome analysis) or Q Exactive mass spectrometer (for analyses of whole proteome and OGA interactome) equipped with a Dionex Ultimate 3000 UPLC system (Thermo Scientific). Peptides were separated on a 75 μm × 15 cm homemade column packed with BEH C18 (1.7 μm, 150 Å, Waters). Mobile phase A consisted of 0.1% FA, and B consisted of ACN/0.1% FA. A linear gradient of 3–30% B in 18 min, 30–75% B in 80 min, and 75–95% B in 10 min was employed throughout the study. For glycosylated peptide analysis on Fusion Lumos, mass spectra from survey full scans were acquired on the Orbitrap (m/z 300–1800) at a resolution of 60,000 with automated gain control (AGC) value of 2×10^5^ and maximum injection time of 50 ms. The data dependent HCD was acquired on the most intense precursor ions at a resolution of 30,000 and a normalized collision energy of 30%. If the biotin fragment ions^60^ derived from the oxonium ion at m/z 270.127 or m/z 420.217 (± m/z 0.01) were detected within the top 10 most abundant peaks, the EThcD collision-induced dissociation MS/MS scan would be triggered with a resolution of 60,000 and a normalized collision energy of 25% at AGC value of 5×10^4^. For other peptide analyses by Q Exactive, mass spectra from survey full scans were acquired on the Orbitrap (m/z 300–1500) at a resolution of 70,000 with AGC value of 10^6^ and maximum injection time of 100 ms. The top 15 precursor ions were selected for the subsequent data dependent HCD MS/MS fragmentation at a resolution of 17,500 and the AGC value of 10^5^. All proteomic analyses were performed with three biological and two technical repeats.

### Proteomic Data Analysis

Raw MS spectra were processed for peak detection and quantitation using MaxQuant^88^ (v. 1.6.1.0) and Perseus^89^ (v. 1.6.7.0) software with default settings. Peptides were searched against the Uniprot database^90^ (release 2017_09). Basic search criteria including trypsin specificity, dimethyl labeling, variable modifications of oxidation (Met) and carbamidomethyl (Cys), and up to two missed cleavages were used for all data processing. Specific variable modifications were applied in O-GlcNAcome dataset: glycosylation of Cys/Ser/Thr (H_47_C_29_N_7_O_11_S, +701.305 Da). Using a decoy database strategy, peptide identification was accepted based on the posterior error probability with a false discovery rate of 1%. Precursor intensities of identified peptides were further searched and recalculated using the “match between runs” option in MaxQuant. For peptides with post-translational modifications (PTMs), the localization probability of all putative modification sites was calculated by the MaxQuant PTM score algorithm based on the peptide spectral match and the potential modification sites in each peptide^91^. Sites with a PTM probability > 0.75 were considered unambiguously identified. Data imputation algorithm incorporated in Perseus was applied to enable statistical evaluation. For the quantitative analysis of O-GlcNAcome, the log_2_ ratio (mutant/WT) of O-GlcNAc site was normalized to the log_2_ ratio of proteins from the whole proteome (proteins not detected in the whole proteome were given log_2_ ratio = 0). Regarding the average standard deviation and the size of dataset from each proteomic analysis, O-GlcNAc sites that displayed a minimum of average 1.5-fold change (*p* ≤ 0.05) between WT OGA and mutant cells were considered dysregulated. For quantitative analysis of the whole proteome and OGA interactome, protein groups with a minimum of average 1.75-fold change (*p* ≤ 0.05) between WT OGA and mutant cells were considered dysregulated. The p-value was calculated by one-tailed paired student t-test using Excel (Microsoft).

### Bioinformatics Analysis

Volcano plot was generated using VolcaNoseR^92^. Information of protein-protein interactions and the functional term enrichment were retrieved from STRING database^93,94^ (STRING consortium, v.1.5.1) with a confidence threshold of 0.5. Visualization of protein association network was conducted by Cytoscape^95^ software (Cytoscape Consortium, v. 3.7.1). Protein clustering was performed using MCL (Markov Clustering) algorithm^96^. Six extra protein nodes were randomly added for network generation to achieve better clustering and connection between proteins. The sequence logo of peptides flanking the O-GlcNAc site was generated by pLogo^97^. O-GlcNAc sites near C- or N-terminus of proteins with the flanking sequence shorter than seven residues were excluded from pLogo analysis. Venn diagram was generated by Free Venn Diagram Marker software (Media Freeware, v. 1.0.0).

### Protein Purification

To purify O-GlcNAcylated PDLIM7 (gPDLIM7), the pgPDLIM7 plasmid was transformed into *E. coli* Rosetta2(DE3) competent cells, and the transformants were grown at 37 °C in Luria-Bertani (LB) media. After the optical density reached 0.6 at 600 nm, the culture was induced by 0.3 mM isopropyl β-D-1-thiogalactopyranoside (IPTG) at 16 °C for 16 h. The cells were pelleted, resuspended in buffer containing 50 mM Na_3_PO_4_ pH 8.0, 300 mM NaCl, and 1 mM phenylmethylsulfonyl fluoride (PMSF). Cells were lysed with ultra-high-pressure cell disruptor EmulsiFlex-C5 (Avestin). After centrifugation, the supernatant was subjected to an Ni-NTA column (Qiagen) for affinity purification. The recombinant protein was subsequently eluted by buffer containing 50 mM Na_3_PO_4_ pH 8.0, 300 mM NaCl, 250 mM imidazole. Further purification was performed by size-exclusion chromatography (Superdex 200 increase 10/300; GE Healthcare) in the buffer containing 50 mM Na_3_PO_4_ pH 8.0, 300 mM NaCl, and 0.5 mM Tris(3-hydroxypropyl)phosphine (THP). The O-GlcNAcylation of purified gPDLIM7 protein was validated by western blot analysis using O-GlcNAc antibody (CTD110.6) as mentioned above.

To purify OGA, the plasmid pET21b-OGA was transformed into *E. coli* BL21(DE3) competent cells, and the transformants were grown at 37 °C in LB media. After optical density reached 0.6 at 600 nm, the culture was induced by 0.3 mM IPTG at 16 °C for 16 h. The cells were pelleted, resuspended in buffer containing 20 mM sodium phosphate pH 7.0, 150 mM NaCl, and 1 mM PMSF. Cell lysis and affinity purification with Ni-NTA column were performed similarly as above. The recombinant protein was eluted with buffer containing 20 mM sodium phosphate pH 7.0, 150 mM NaCl, and 250 mM imidazole. The eluted OGA was further purified by size-exclusion chromatography in buffer containing 20 mM sodium phosphate pH 7.0, 150 mM NaCl, and 0.5 mM THP. OGA mutant proteins were expressed and purified similarly. All purified proteins were stored at −80 °C until use.

### OGA Enzymatic Assay

The steady-state kinetics of WT OGA and mutants were measured with the fluorogenic substrate 4-methylumbelliferyl-N-acetyl-β-D-glucosaminide (4MU-GlcNAc) as previously reported^57^. Briefly, 2 nM enzyme was incubated with each of 10, 50, 150, 300, 600, and 1200 μM 4MU-GlcNAc as the substrate in 25 μL reaction (50 mM NaH_2_PO_4_ pH 6.5, 100 mM NaCl, 0.1 mg/mL BSA) for 10 min at 37 °C. The reaction was quenched by 150 μL of 200 mM glycine pH 10.75. The fluorescence of liberated 4-methylumbelliferone was measured using BioTek Synergy H1 Hybrid microplate reader (Agilent) with excitation and emission wavelengths of 360 and 450 nm, respectively. Enzymatic activity was converted to μM/min using a standard curve of free 4-methylumbelliferone. All analyses were carried out in triplicate. Michaelis-Menten kinetic parameters were calculated by Prism 5 software (GraphPad).

### *In vitro* OGA Deglycosylation Assay

Recombinantly purified gPDLIM7 protein (5 μM) was mixed with 1 μM recombinantly purified WT OGA or mutant in a 10 μL reaction containing 50 mM sodium phosphate pH 8.0, 250 mM NaCl, and 0.5 mM THP. The reaction was conducted at 37 °C for 30 min with gentle rotation. One sample incubated with 10 μM thiamet-G was used as a negative control. The reaction was quenched by SDS-loading buffer, boiled, and subjected to SDS-PAGE for western blot analysis as mentioned above.

### Cell Growth and Anchorage-Independent Soft Agar Assays

To measure the cell growth with 3-(4,5-dimethylthiazol-2-yl)-2,5-diphenyltetrazolium bromide (MTT), T-REx-293 cells with inducible WT OGA or mutant were cultured in 96-well plates at 30% confluency (100 xL/well) in DMEM supplemented with 10% FBS (tetracycline-free, Takara Bio) 24 h prior to doxycycline induction. Cell viability was measured at 0 h and the indicated time point post-induction by adding 20 μL of MTT to each well. After 3 h of incubation at 37 °C, the formazan dye formed in intact cells was solubilized by isopropanol containing 40 mM HCl and measured at 560 nm with background subtraction at 670 nm on BioTek Synergy H1 Hybrid microplate reader. Cell density measured at the indicated post-induction time was normalized to the density at 0 h. Results from nine wells of each condition were used for quantitation. For resazurin assay, cells with doxycycline induction were similarly prepared as above. Cells were then incubated with 50 μg/mL of resazurin for 4 h at 37 °C. Cell density was then measured on the plate reader with an excitation and emission wavelength of 544 and 590 nm, respectively. Data from four wells were used for quantitation.

For soft agar assay, cells were cultured in DMEM supplemented with 10% FBS (tetracycline-free) in 6-well plates at 60% confluency. After 48 h of doxycycline induction, cells were trypsinized and mixed with 0.48% low-melt point agarose in culture media, and then plated on top of the base layer of media containing 0.8% agarose in 6-well plates. The agarose layer was covered with 1 mL of growth media (+/− doxycycline) and replaced every 3 d. After 3–4 weeks, the colonies were dyed by crystal violet (0.005%) with 4% formaldehyde and imaged by C600 imaging system. The total colony density of each well was quantified by Image J (v 1.48, Public Domain) or AzureSpot (v 2.2.167, Azure Biosystem) software.

### Wound Healing Assay

Cells were seeded in 6-well plates after 48 h of doxycycline induction and reached 100% confluency before wound making. The scratch wounds were made using a sterile 200 μL pipette tip. Doxycycline was added in the media during healing if needed. At 0 and 24 h post-scratching, cells were imaged using an inverted microscope AE2000 (Motic) at 100x magnification with the ocular lens attached to a digital camera. The wound area at 0 and 24 h post-scratching was quantified by Image J.

### RNA Extraction and cDNA Synthesis

For RNA extraction, cells were cultured in 6-well plates at 30–40% confluency. After 48 h of doxycycline induction, RNA purification was performed using TRIzol reagent (Invitrogen). 1.5 μg RNA with Oligo(dT)_18_ and random hexamer primers were used for cDNA synthesis. cDNA synthesis was conducted by Maxima H Minus First Strand cDNA Synthesis Kit with DNase (Life Technology) or GoScript^™^ Reverse Transcriptase kit (Promega) according to manufacturer’s instructions. cDNA samples were stored at −80 °C and used for real-time qPCR analysis within one week.

### Real-Time qPCR for p53 Transcription Analysis

The PCR sample was prepared using 2 ng cDNA and PowerUp SYBR Green Master Mix (Life Technology) following manufacturer’s instruction. No-template reaction was included as negative control. The forward and reverse primers used for p53 and reference gene GAPDH were listed in **Supplementary Table 8**. Real-time qPCR was performed using StepOne Plus Real-Time PCR system (Applied Biosystems) with the following cycling parameters: 50 °C for 2 min, 95 °C for 2 min, 40 cycles of amplification at 95 °C for 15 sec, 55 °C for 30 sec, and 72 °C for 1 min. The calculation of cycle threshold (Cт) values was performed using StepOne software (v. 2.1, Applied Biosystems). Each sample was assessed with four to five technical repeats. The relative abundance of the transcripts was analyzed by 2^−ΔΔCт^ method as previously reported^98^.

### In-Cell Ubiquitination Assay

For cell lines with stable or inducible expression of cMyc-tagged WT PDLIM7 or mutant, cells were co-transfected with HA-tagged p53 and ubiquitin. For other cell lines, cells were co-transfected with plasmids containing HA-tagged p53, cMyc-tagged WT PDLIM7 or mutant, and ubiquitin as mentioned above. The culture medium was changed to fresh medium 24 h post-transfection. Doxycycline was also added to the media if the induction of protein expression or endogenous protein knockdown was needed. After culturing for another 24 h, medium was changed again to the one containing 35 μM MG132 (1% DMSO, +/− doxycycline). Cells were then incubated for another 3 h prior to harvesting. For cells with MDM2-p53 inhibition, 20 μM Nutlin-3a (0.5% DMSO, +/− doxycycline) was added to the cells 12 h prior to MG132 treatment. Cells were then washed by PBS, harvested by scrapping, snap-frozen in liquid N_2_, and stored at −80 °C until use.

### Analysis of Global and PDLIM7 Phosphorylation

To analyze the global phosphoproteins, HEK293 cells were prepared following the procedure of in-cell ubiquitination assay. The whole cell lysates were then prepared and separated by SDS-PAGE as mentioned above. Phosphoproteins were stained using Pro-Q Diamond Phosphoprotein Gel Stain following manufacturer’s instructions (Life Technology) and detected by fluorescence scanning. The Coomassie blue staining was then applied to obtain the total protein loading. All imaging was performed on C600 imaging system.

For analysis of PDLIM7 phosphorylation, the immunoprecipitated cMyc-tagged WT PDLIM7 or mutant was lysed, digested, and dimethyl labeled as mentioned above. Phosphopeptides were enriched by immobilized metal affinity chromatography (IMAC) as previously reported^99^. Briefly, to freshly prepared IMAC slurry, Ni-NTA Superflow agarose (Qiagen) was stripped with 100 mM EDTA for 30 min, reloaded with 10 mM FeCl_3_ for 30 min, washed three times with HPLC water, and resuspended in IMAC solution (1:1:1:1, beads/ACN/MeOH/0.01% acetic acid, v/v/v/v). The dimethyl labeled peptides were resuspended in final 80% ACN/0.1% FA and incubated with IMAC beads for 30 min at r.t. with rotation. The beads were then washed with 80% ACN/0.1% FA and 0.1% FA. The phosphopeptides were eluted by 500 mM potassium phosphate buffer followed by C18 StageTip desalting. The final peptide samples were dried by vacuum centrifugation and stored at −80 °C. The peptide samples without phosphopeptide enrichment were used to analyze the total protein level. The nanoLC-MS/MS analysis of phosphopeptides was performed on Q Exactive mass spectrometer similarly as mentioned above. Data analysis and phosphopeptide quantitation were conducted by the aforementioned MaxQuant with phosphorylation of Ser/Thr/Tyr as a specific variable modification.

### Statistical Analysis

The statistical analysis of data from cell growth, anchorage-independent growth, wound healing, and western blot were performed by Excel or Prism 5 software. Data comparison was analyzed by one-tailed paired or unpaired student t-test; n ^3^ 3. All error bars denote the standard deviation. The statistical significance cutoff was set at *p* < 0.05.

## Data Availability

The MS/MS proteomic data and output tables of MaxQuant analysis have been deposited to the ProteomeXChange Consortium (http://proteomecentral.proteomexchange.org) via the PRIDE partner repository^100^. The dataset identifiers are PXD039798 (O-GlcNAcome), PXD039800 (whole proteome), and PXD039801 (OGA interactome).
